# Sensitivity to Lysosome-Dependent Cell Death Is Directly Regulated by Lysosomal Cholesterol Content

**DOI:** 10.1371/journal.pone.0050262

**Published:** 2012-11-16

**Authors:** Hanna Appelqvist, Linnea Sandin, Karin Björnström, Paul Saftig, Brett Garner, Karin Öllinger, Katarina Kågedal

**Affiliations:** 1 Experimental Pathology, Department of Clinical and Experimental Medicine, Faculty of Health Sciences, Linköping University, Linköping, Sweden; 2 Anaesthesiology, Department of Medical and Health Sciences, Faculty of Health Sciences, Linköping University, Linköping, Sweden; 3 Department of Anaesthesiology UHL, County Council of Östergötland, Linköping, Sweden; 4 Biochemical Institute, Christian-Albrechts-University Kiel, Kiel, Germany; 5 Illawarra Health and Medical Research Institute, University of Wollongong, Wollongong, Australia; 6 School of Biological Sciences, University of Wollongong, Wollongong, Australia; 7 Department of Clinical Pathology and Clinical Genetics, County Council of Östergötland, Linköping, Sweden; Foundation for Biomedical Research Academy of Athens, Greece

## Abstract

Alterations in lipid homeostasis are implicated in several neurodegenerative diseases, although the mechanisms responsible are poorly understood. We evaluated the impact of cholesterol accumulation, induced by U18666A, quinacrine or mutations in the cholesterol transporting Niemann-Pick disease type C1 (NPC1) protein, on lysosomal stability and sensitivity to lysosome-mediated cell death. We found that neurons with lysosomal cholesterol accumulation were protected from oxidative stress-induced apoptosis. In addition, human fibroblasts with cholesterol-loaded lysosomes showed higher lysosomal membrane stability than controls. Previous studies have shown that cholesterol accumulation is accompanied by the storage of lipids such as sphingomyelin, glycosphingolipids and sphingosine and an up regulation of lysosomal associated membrane protein-2 (LAMP-2), which may also influence lysosomal stability. However, in this study the use of myriocin and LAMP deficient fibroblasts excluded these factors as responsible for the rescuing effect and instead suggested that primarily lysosomal cholesterol content determineD the cellular sensitivity to toxic insults. Further strengthening this concept, depletion of cholesterol using methyl-β-cyclodextrin or 25-hydroxycholesterol decreased the stability of lysosomes and cells became more prone to undergo apoptosis. In conclusion, cholesterol content regulated lysosomal membrane permeabilization and thereby influenced cell death sensitivity. Our data suggests that lysosomal cholesterol modulation might be used as a therapeutic strategy for conditions associated with accelerated or repressed apoptosis.

## Introduction

Lysosomes are acidic organelles involved in several cellular functions, including degradation of macromolecules, repair of the plasma membrane, antigen presentation, recycling of cell surface receptors and apoptosis signaling [1]. Upon a variety of cell death stimuli, lysosomal membrane permeabilization (LMP) is induced and this results in the release of lysosomal content to the cytosol. Previous studies have convincingly shown that the presence of lysosomal proteases, cathepsins, in the cytosol mediates apoptosis [2], [3], [4], implying that the integrity of the lysosomal membrane is of high importance for cell survival. The mechanism underlying LMP is still incompletely understood; however, a number of factors have been described to affect the stability of the lysosomal membrane, including the level of lysosome-associated membrane proteins (LAMP) and cholesterol [5].

Niemann-Pick disease type C (NPC) is a complex neurodegenerative lysosomal storage disorder caused by mutations in the genes encoding the cholesterol transporting proteins NPC1 and NPC2. Normally, cholesterol is released from endocytosed low density lipoprotein (LDL) particles by the action of lysosomal acid lipase and is then transported, via the lysosomal NPC proteins, to the ER where it serves as a sensor for cellular cholesterol homeostasis and may be esterified [6]. Nonfunctional NPC proteins disturb cholesterol efflux from the lysosomes. Thus, NPC-mutated cells are characterized by the accumulation of unesterified cholesterol in the endo-lysosomal system [7]. Other lipids, including sphingomyelin, glycosphingolipids, sphingosine and bis(monoacylglycero)phosphate (BMP) accumulate in the lysosomes in NPC as well [8], [9]. At present there is no cure for NPC, and the goal for therapeutic treatment is to diminish the lipid load. Alleviation of the NPC phenotype can be obtained by several approaches, e.g., by decreasing cholesterol levels [10], inhibiting glycosphingolipid synthesis [11] or increasing lipid degradation [12].

β-Cyclodextrin compounds has been shown to correct cholesterol transport in NPC-defective cells [13] and substantially reduce neurodegeneration and increase lifespan in Npc1^−/−^ mice [14]. Several substances have the ability to decrease lysosomal cholesterol; for example, 25-hydroxycholesterol (25-HC) down-regulates cholesterol accumulation through homeostatic ER mechanisms by signaling cholesterol excess [15]. Lipidosis, and intracellular accumulation of phospholipids, is a side effect of certain cationic amphiphilic drugs, including quinacrine, desipramine, imipramine and amiodarone, used to treat e.g., depression and arrhythmias [16], [17]. The exact mechanism of action of these small lysosomotropic compounds remains poorly understood, but their amphiphilic nature allows them to accumulate in membranes and might disrupt the activity of membrane proteins like NPC1 [18]. In addition, the compound U18666A has been extensively used to mimic the NPC phenotype by impairing the intracellular transport of LDL-derived cholesterol from lysosomes [19], thus resulting in cholesterol accumulation in this compartment.

The way in which increased lysosomal cholesterol contributes to NPC is unknown, but it has been suggested that both lipid storage and a concomitant inflammatory response, involving macrophages in peripheral organs and activated glia in the central nervous system, converge to produce the pathological lesions that characterize the disease [10]. Recently, we reported that enhanced lysosomal cholesterol content protects cells from LMP-dependent apoptosis [20]. Although this finding, which has also been confirmed by others [21], may seem counterintuitive, it is possible that cholesterol preserves the integrity of the lysosomal membrane and thus promotes neuronal survival upon acute cellular stress. Importantly, in both NPC1-mutant cells and U18666A treated cells, cholesterol accumulation is associated with storage of several other lipids [9], [22], which might influence lysosomal stability. In addition, the expression of LAMP-2 was increased by U18666A treatment [20]. Because LAMPs have been shown to be important for regulation of LMP [23], further studies to distinguish between the LMP-modulating roles of cholesterol, sphingolipids and altered LAMP-1 and −2 expression were undertaken. We hypothesize that modulation of lysosomal composition affects cellular sensitivity to apoptosis and cell fate can be manipulated by the use of agents inducing or reducing cholesterol content. We herein provide evidence that cholesterol, and not accompanying sphingolipids or LAMP proteins, stabilizes lysosomes and thereby protects from cell death.

## Results

### Treatment with cholesterol modifying drugs results in alterations of the lysosomal compartment

**Figure 1:**
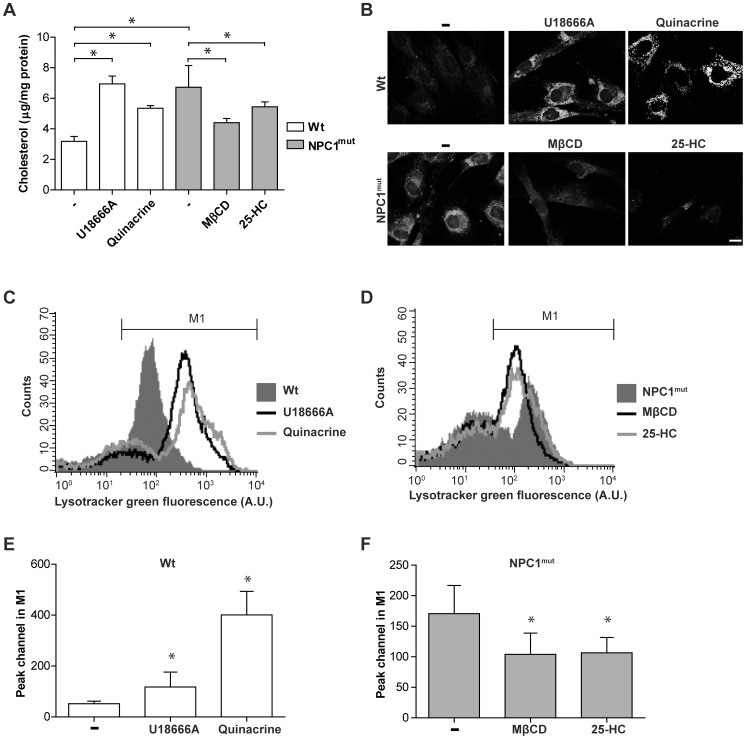
Cholesterol modulation in human fibroblasts is associated with alterations of the lysosomal compartment. Human wt fibroblasts were treated with U18666A or quinacrine to induce cholesterol accumulation, and NPC1-mutant fibroblasts were treated with methyl-β-cyclodextrin (MβCD) or 25-hydroxy cholesterol (25-HC) to revert cholesterol storage. **A**) Measurement of unesterified cholesterol (n = 4) and **B**) representative images of filipin staining (scale bar 10 µm). **C and D**) Representative histogram from flow cytometric analysis of Lysotracker fluorescence staining. M1 gate denotes the highly fluorescent population. **E** and **F**) Quantification of peak channel in the M1 population (seen in C and D; n = 4). Data are presented as the mean ± SD, * p≤0.05.

We hypothesized that modulation of lysosomal composition affects cellular sensitivity to apoptosis and tested our theory using human fibroblasts derived from a patient with NPC. The cells harbor mutations in the NPC1 gene and have a negligible expression of the NPC1 protein (results not shown). Cellular cholesterol levels were 2-fold higher in NPC1-mutant fibroblasts compared with wild type (wt) fibroblasts (Figure 1A). Labeling of unesterified cholesterol using the antibiotic filipin showed a perinuclear vesicular pattern in NPC1-mutant cells, confirming that cholesterol accumulated in late endosomes/lysosomes (Figure 1B). Cholesterol levels were manipulated in these two cell models, by agents that were reported to change the lipid composition of lysosomes. Treatment of wt fibroblasts with U18666A or quinacrine resulted in cholesterol accumulation, as assessed by analysis of unesterified cholesterol content and lysosomal location was confirmed by filipin staining (Figure 1A and B). In addition, both agents induced expansion of the lysosomal compartment, as demonstrated by increased Lysotracker fluorescence (Figure 1C and E). NPC1-mutant fibroblasts were treated with methyl-β-cyclodextrin (MβCD) and 25-HC, substances suggested to revert cholesterol storage. The results verified that both agents significantly reduced cholesterol content and decreased filipin fluorescence (Figure 1A and B). Both drugs reduced Lysotracker fluorescence of NPC1-mutant fibroblasts (Figure 1D and F), indicating that reversion of cholesterol load is accompanied by normalization of the lysosomal compartment.

### Cholesterol content influences lysosomal stability and affects apoptosis sensitivity

**Figure 2:**
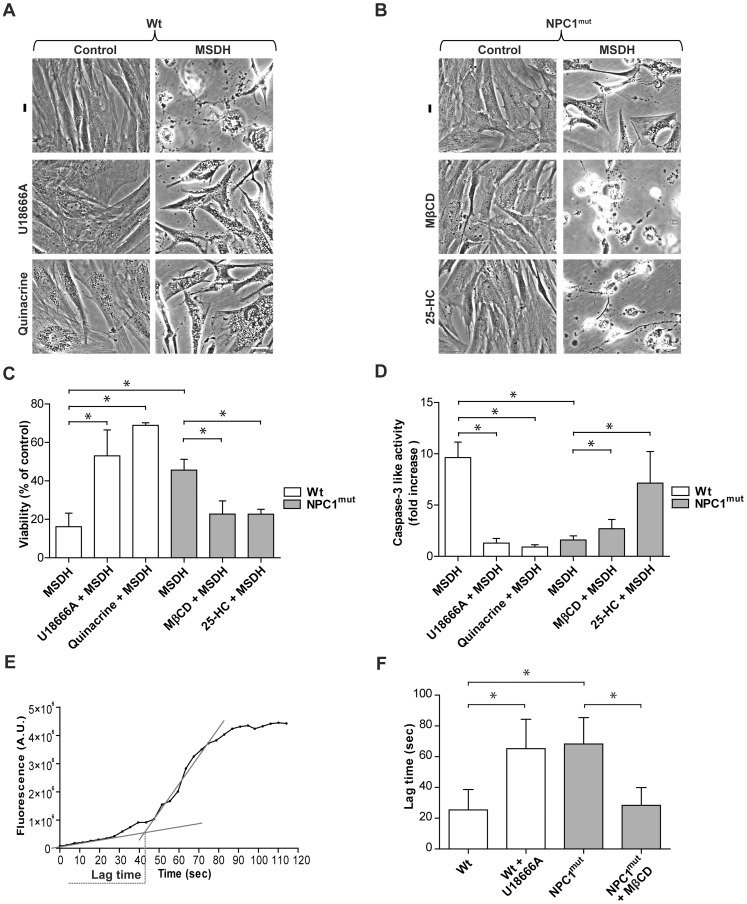
Manipulation of lysosomal cholesterol content modulates the cellular sensitivity to apoptosis. Cholesterol content of human fibroblasts was modulated using U18666A, quinacrine, methyl-β-cyclodextrin (MβCD) or 25-hydroxy cholesterol (25-HC) before apoptosis was induced using O-methyl-serine dodecylamide hydrochloride (MSDH; 24 h). Phase contrast images of **A**) wt and **B**) NPC1-mutant fibroblasts (NPC1^mut^). Scale bar 20 µm. **C**) Viability of cultures in A and B, respectively, assessed by the MTT assay (n = 4). Viability is expressed as percentage of untreated cultures. **D**) Caspase-3 like activity (n = 4–8). **E**) Representative curve of increase in green fluorescence during photo-oxidation of acridine orange. **F**) Quantification of lag time (as presented in E; n = 5–6). Data are presented as the mean ± SD, * p≤0.05.

To investigate whether lysosomal cholesterol content could be involved in lysosomal stability and thereby affect the cellular sensitivity to apoptosis, wt and NPC1-mutant fibroblasts were exposed to O-methyl-serine dodecylamide hydrochloride (MSDH), a lysosomotropic detergent previously demonstrated to induce apoptosis via LMP [20], [24]. MSDH induced a substantial loss of viability in wt fibroblasts, while NPC1-mutant fibroblasts were less sensitive (Figure 2A–C). Treatment of wt fibroblasts with U18666A or quinacrine to increase lysosomal cholesterol content prior to exposure to MSDH significantly decreased the sensitivity to apoptosis induction (Figure 2A and C). Conversely, cholesterol reduction in NPC1-mutant fibroblasts, induced either by MβCD or 25-HC pretreatment, increased the sensitivity to cell death (Figure 2B and C), which further indicates that cholesterol has a cytoprotective effect. Results obtained by the MTT viability assay (Figure 2C) were verified by caspase-3 activity measurement (Figure 2D) and crystal violet staining (Figure S1). This confirms that cholesterol is an important factor in the regulation of apoptosis.

Photo-oxidation of acridine orange (AO), was applied to analyze lysosomal membrane stability as previously described [25]. The weak base AO accumulates in lysosomes and lysosomal rupture can be enforced by exposure to blue light. The loss of lysosomal integrity was measured as a distinct increase in AO-fluorescence in the cytosol, and the lag time, from the start of laser irradiation until rupture of lysosomes, was estimated (Figure 2E). NPC1-mutant cells showed a longer lag time before lysosomal rupture compared to wt cells (Figure 2F). Similarly, wt cells treated with U18666A showed a longer lag time before lysosomal rupture compared to untreated control wt cells (Figure 2F). This indicates that cells with cholesterol accumulation have a more stable lysosomal membrane. In addition, treatment of NPC1-mutant cells with the cholesterol reducing agent MβCD resulted in a shorter lag time before lysosomal rupture (Figure 2F), which is consistent with decreased lysosomal membrane stability. These results indicate that cholesterol regulates apoptosis sensitivity at the level of LMP and is not a result of perturbation of up- or downstream signaling.

### Myriocin decreases the level of sphingomyelin in human fibroblasts but does not affect cell death sensitivity

**Figure 3:**
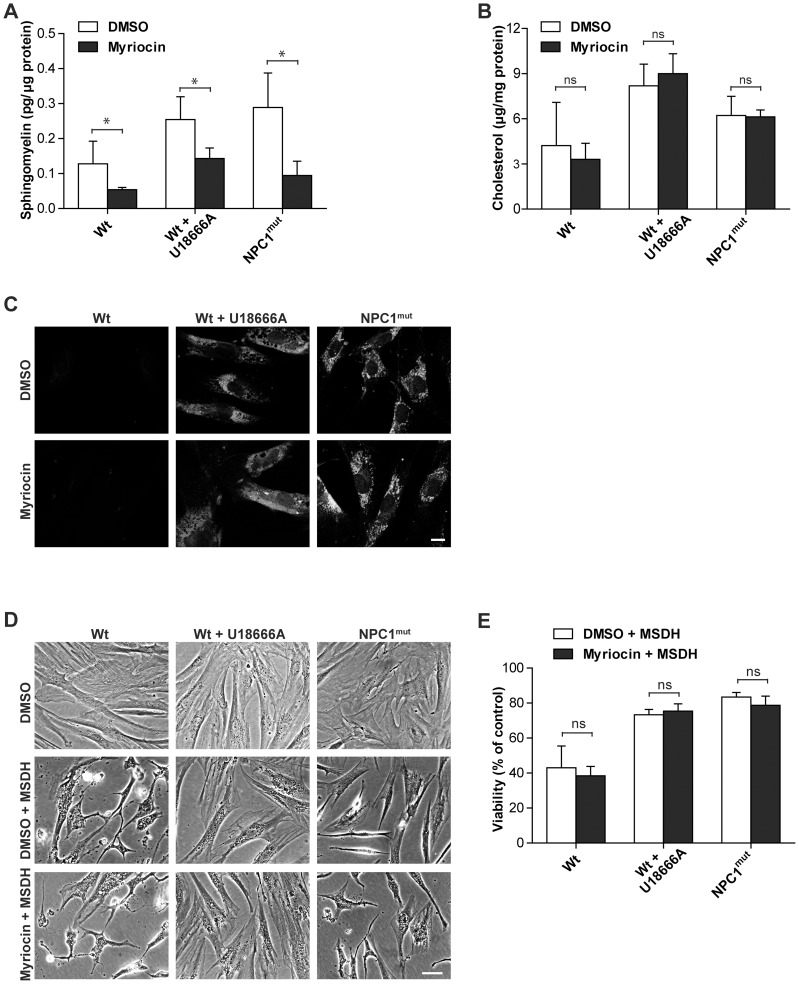
Cholesterol, and not accumulating sphingolipids, is responsible for the apoptosis protection. Human wt fibroblasts, with or without U18666A treatment, and NPC1-mutant fibroblasts were treated with vehicle (dimethyl sulfoxide; DMSO) or myriocin to inhibit sphingolipid biosynthesis. **A**) Sphingomyelin (n = 3), **B**) cholesterol content (n = 4) and **C**) filipin staining (scale bar 10 μm) of human fibroblasts. **D**) Phase contrast images of human fibroblasts exposed to O-methyl-serine dodecylamide hydrochloride (MSDH; 24 h). Scale bar 20 μm. **E**) Viability of cultures in D, assessed by the MTT assay (n = 3). Viability is expressed as percentage of MSDH-untreated cultures. Data are presented as the mean ± SD, * p≤0.05, ns; non-significant.

In addition to cholesterol, both NPC1-deficient cells and U18666A-treated cells accumulate several other lipids, including sphingomyelin, glycosphingolipids and sphingosine [9], [22], which have been suggested to influence the stability of lysosomes [26], [27]. By employing myriocin, an inhibitor of serine palmitoyltransferase, which catalyzes the initial step in sphingolipid biosynthesis, the levels of sphingomyelin, sphingosine and glycosphingolipids are all reduced [28]. Sphingomyelin is the major product of the sphingolipid biosynthetic pathway, and spectrophotometric analysis of myriocin-treated wt fibroblasts (with or without U18666A-treatment) and NPC1-mutant fibroblasts confirmed that myriocin was able to decrease the amount of sphingomyelin in these cells by at least 40% (Figure 3A). Of note, in a similar experimental setting filipin staining was demonstrated to be diminished by prolonged myriocin treatment [22]. However, control experiments verified that cholesterol content was not affected by myriocin treatment (Figure 3B and C). Moreover, treatment with myriocin in our experimental model did not change the sensitivity of cells to MSDH-induced apoptosis (Figure 3D and E). These results were verified by crystal violet staining (data not shown). Thus, reducing sphingolipids in cells that maintain lysosomal cholesterol accumulation does not affect LMP-induced cell death.

### Lysosomal cholesterol accumulation protects cortical neurons from apoptosis

**Figure 4:**
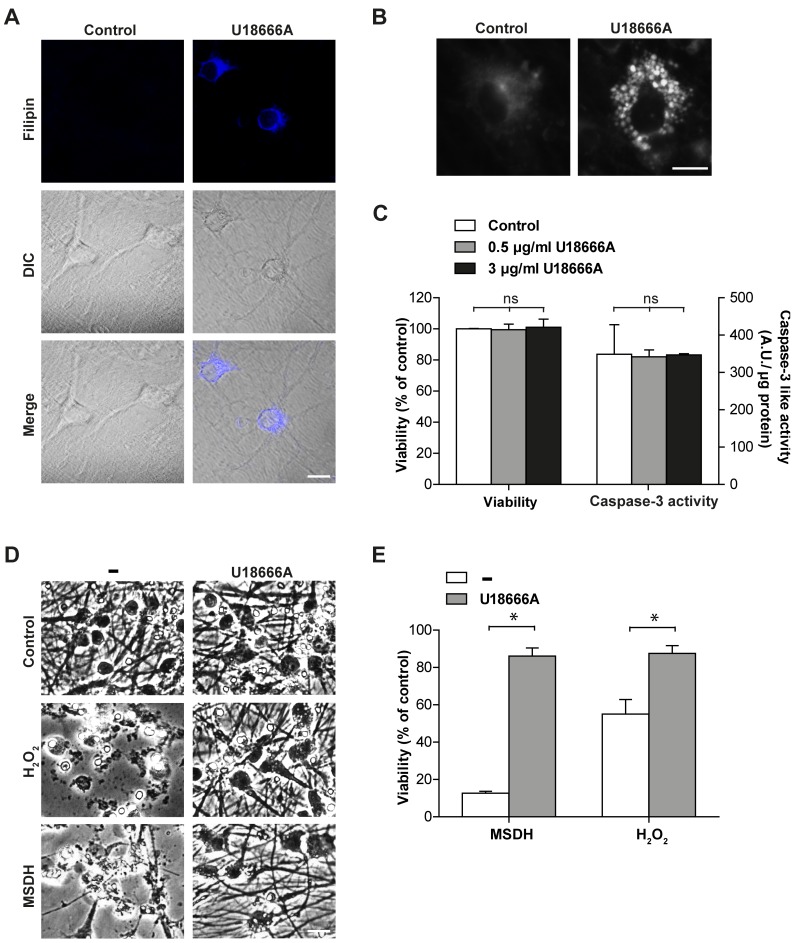
Cholesterol Accumulation in Cortical Neurons Rescues Cells from Apoptosis Induced by MSDH and Oxidative Stress. Cortical neurons were treated with U18666A. **A**) Filipin staining and differential interference contrast microscopy (DIC) images (scale bar 10 µm) and **B**) a higher magnification of filipin staining (scale bar 10 μM). **C**) Viability analysis and caspase-3-like activity (n = 3) after 72 h. **D**) Phase contrast images (scale bar 20 µm) and **E**) viability analysis (MTT assay; n = 3) of cultures exposed to O-methyl-serine dodecylamide hydrochloride (MSDH) or H_2_O_2_, generated by glucose oxidase, with or without pretreatment with U18666A (48 h). Viability is expressed as percentage of untreated control. Data are presented as the mean ± SD, * p≤0.05, ns; non-significant.

Because NPC disease is characterized by neuronal cell death and U18666A has previously been demonstrated to be toxic to neurons [29], we tested the effect of cholesterol accumulation in primary cortical rat neurons. U18666A treatment induced cholesterol redistribution into the endolysosomal system in the neurons as evident from filipin staining (Figure 4A and B), but there was no net increase in cellular cholesterol content (Figure S2). The lysosomal cholesterol accumulation induced by U18666A was non-toxic, as neither loss of viability nor activation of caspase-3 was observed, even at concentrations up to 3 μg/ml (Figure 4C). Importantly, U18666A-induced cholesterol accumulation protected neurons from the lysosome-dependent cell death induced by MSDH (Figure 4D and E). As oxidative stress is a common feature of many diseases affecting the brain, the sensitivity of neurons to H_2_O_2_-induced apoptosis was also investigated. Cholesterol-loaded neurons were less sensitive to oxidative stress-induced apoptosis as well (Figure 4D and E).

### Cholesterol protects MEFs from apoptosis independent of LAMP expression

**Figure 5:**
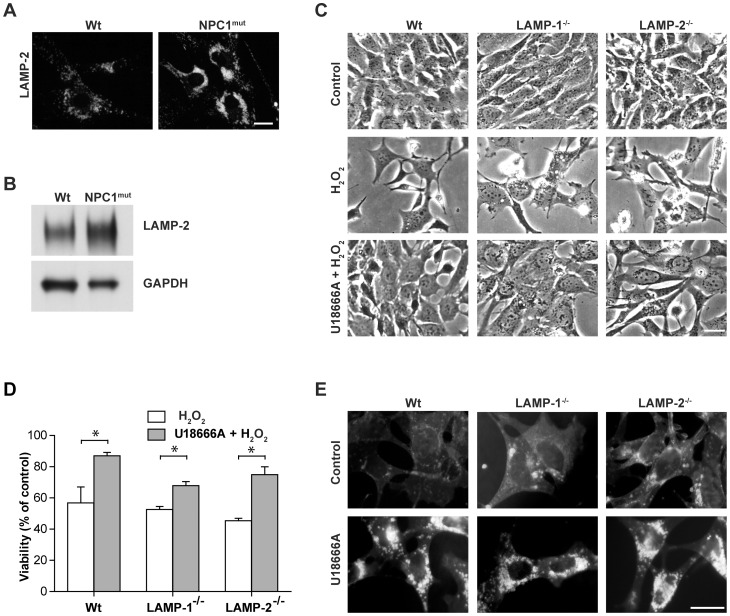
Cholesterol Accumulation Protects MEFs from Oxidative Stress-induced Apoptosis, Independent of the Expression of LAMP Proteins. **A**) Localization (scale bar 10 μm) and **B**) expression of lysosome-associated membrane protein-2 (LAMP-2) in wt and NPC1-mutant (NPC1^mut^) human fibroblasts. Glyceraldehyde-3-phosphate dehydrogenase (GAPDH) was used to verify equal protein loading. One representative blot out of three is shown. **C**) Phase contrast images (scale bar 5 µm) and **D**) viability analysis (n = 4) of wt mouse embryonic fibroblasts (MEFs) and MEFs deficient for LAMP-1 (LAMP-1^−/−^) or LAMP-2 (LAMP-2^−/−^) 24 h after H_2_O_2_ exposure, with or without U18666A pretreatment. Viability was measured by crystal violet staining and expressed as percentage of untreated cultures. Data are presented as the mean ± SD, * p≤0.05) Filipin staining in MEFs, with or without U18666A treatment. Scale bar 10 µm.

**Figure 6:**
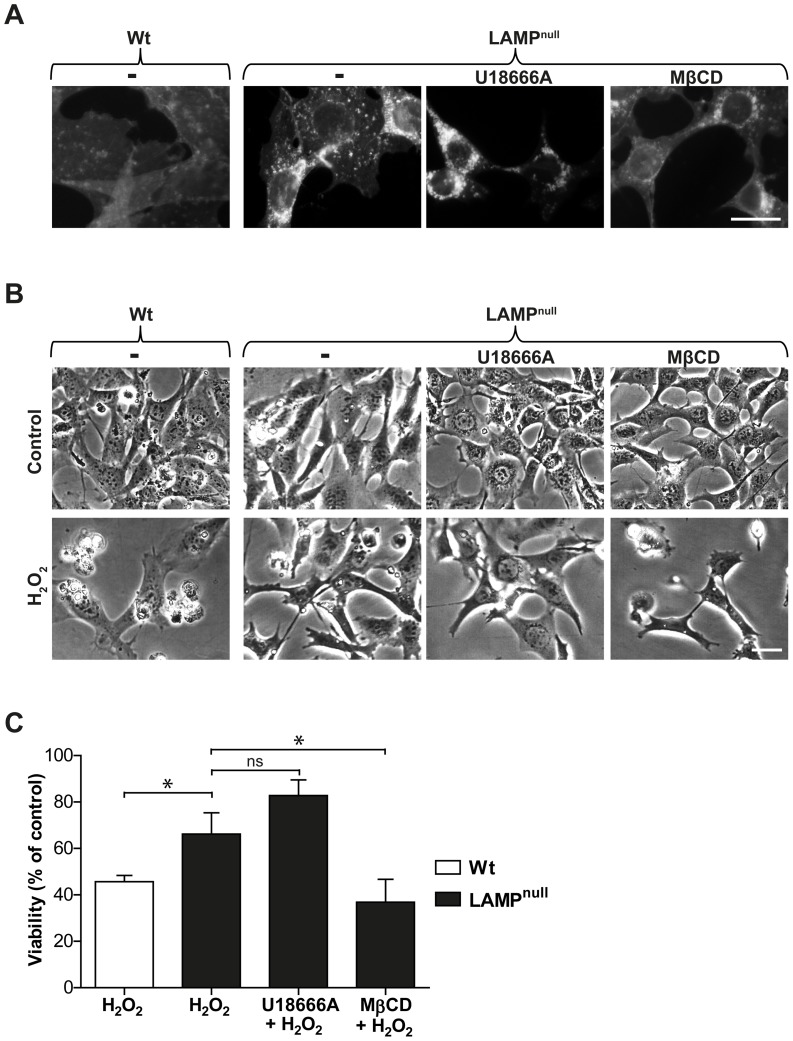
Cholesterol Modulation Influences the Sensitivity of MEFs to Oxidative Stress-induced Apoptosis. Mouse embryonic fibroblasts (MEFs) deficient for both LAMP-1 and LAMP-2 (LAMP^null^) were treated with U18666A or methyl-β-cyclodextrin (MβCD). **A**) Filipin staining of wt and LAMP^null^ MEFs (scale bar 10 µm). **B**) Phase contrast images (scale bar 5 µm) and **C**) viability (n = 4) of wt and LAMP^null^ MEFs 24 h after H_2_O_2_ exposure. Viability was measured by crystal violet staining and expressed as percentage of untreated cultures. Data are presented as the mean ± SD, * p≤0.05, ns; non-significant.

NPC1-mutant cells have an increased expression of LAMP-2 compared to wt fibroblasts (Figure 5A and B), and LAMP proteins have recently been suggested to regulate the stability of the lysosomal membrane [23]. Therefore, we decided to investigate the importance of LAMP proteins and took advantage of MEFs deficient for either LAMP-1 (LAMP-1^−/−^) or −2 (LAMP-2^−/−^). Cultures were exposed to oxidative stress induced by H_2_O_2_ addition, and a viability analysis demonstrated that none of the MEF variants displayed any significant difference in sensitivity to cell death compared to wt MEFs (Figure 5C and D). In concordance, LAMP expression did not influence lysosomal stability in MEFs, as there were no significant differences in the lag times until lysosomal destabilization after photo-oxidation of the different cell types (data not shown). U18666A treatment rescued wt, LAMP-1^−/−^ and LAMP-2^−/−^ cells from oxidative stress-induced apoptosis (Figure 5C and D), indicating that LAMP expression is not required for the protective effect of U18666A treatment. Filipin staining verified that untreated wt, LAMP-1^−/−^ and LAMP-2^−/−^ cells had relatively weak and diffuse staining, whereas cells treated with U18666A exhibited increased perinuclear vesicular filipin staining (Figure 5E). In contrast to MEFs deficient for either LAMP-1 or LAMP-2, MEFs deficient for both LAMP-1 and −2 (LAMP^null^) displayed prominent, inherent cholesterol accumulation (Figure 6A), in agreement with an earlier study [30]. Analysis of cholesterol content demonstrated that LAMP^null^ cells contained a significantly higher amount of unesterified cholesterol compared to wt MEFs (13.0±1.8 vs. 8.8±2.0 μg cholesterol/mg protein; p≤0.05), while cells deficient for either LAMP-1 or LAMP-2 did not differ from wt cells. Moreover, LAMP^null^ cells demonstrated a lower sensitivity than wt MEFs to H_2_O_2_-induced cell death (Figure 6B and C). U18666A treatment did not change the cholesterol content, as shown by filipin staining of LAMP^null^ MEFs. This explains why the oxidative stress sensitivity of LAMP^null^ cells was not altered by U18666A pre-treatment (Figure 6A–C). In contrast to U18666A treatment or NPC1 mutation, cholesterol accumulation in LAMP^null^ MEFs is not accompanied by the storage of other lipids [31]. Therefore, in these cells, neither sphingolipids nor LAMP proteins could influence lysosomal stability. Finally, we reduced the cholesterol content of LAMP^null^ cells by MβCD pre-treatment. Such treatment reduced filipin staining and sensitized cells to H_2_O_2_-induced apoptosis (Figure 6A–C). Thus, we confirm that cholesterol accumulation protects cells from apoptosis, and the potential protective effects of accompanying lipids can be excluded.

## Discussion

In this study we have demonstrated that cholesterol accumulation stabilizes lysosomes and confers protection from acute toxic insults induced by a lysosomotropic detergent, photo-oxidation or oxidative stress. We provide novel mechanistic insights by showing that neither sphingolipids, known to accumulate together with cholesterol in lysosomes, nor LAMP proteins are involved in this protective activity. A recent study suggested that unesterified cholesterol modulates cellular susceptibility to ROS-induced LMP by providing an alternative target for oxidants, thus lowering the probability of damage to other lysosomal components [21]. Our data regarding H_2_O_2_ exposure is consistent with this idea. However, because our current study shows that cholesterol also confers protection in cells exposed to the lysosomotropic compound MSDH, although MSDH does not appear to induce ROS production [32], an alternative explanation is that the higher cholesterol content alters the architecture of the lysosomal membrane, making it less sensitive to the effect of the lysosomotropic detergent or oxidants. In our study, lysosomal cholesterol levels were also shown to influence the sensitivity of lysosomes to photo-oxidation. LAMP expression did, however, not influence the stability of lysosomes in our experimental system, although it was previously demonstrated that knockdown of either LAMP-1 or LAMP-2 is sufficient to sensitize cells to photo-oxidation-induced lysosomal destabilization [23]. LAMP-1 and −2 are estimated to constitute approximately 50% of all lysosomal membrane proteins [33]. Jäättelä and colleagues showed that down-regulation of LAMP proteins in human cancer cells sensitizes them to lysosomal cell death pathways induced by various anticancer drugs, indicating that LAMP proteins protect the lysosomal membrane [23]. Knockdown of either LAMP-1 or LAMP-2 was sufficient to sensitize cells to LMP in their experimental model. We found increased expression of LAMP proteins in NPC-deficient cells in this study and in U18666A-treated cells [20]. It is possible that the increased expression of LAMP could contribute to the increased lysosomal stability observed in these cells. However, the lack of LAMP proteins did not significantly alter the sensitivity to oxidative stress-induced apoptosis or photo-oxidation in MEFs, whereas changes in lysosomal cholesterol had a profound effect.

As cholesterol is an important component of all cellular membranes, including specialized lipid raft micro domains [34], modulation of cholesterol content has the ability to induce major changes in cell function. We suggest that cholesterol has an important additional role in the regulation of apoptosis sensitivity by acting at the level of permeabilization of the lysosome. In concordance with our results, Reiners *et al.* conclude that U18666A, as well as imipramine, suppresses apoptosis by inhibiting LMP [21]. We show that alterations in cholesterol load influences cellular sensitivity to MSDH- and oxidative stress-induced apoptosis. MSDH is an agent that specifically targets the lysosomal membrane and is therefore appropriate for studies of lysosomal membrane stability. Because MSDH is an unconventional apoptosis inducer, we have shown in earlier studies that lysosomal cholesterol also protects cells from death caused by the classical apoptosis inducers staurosporine and cisplatin [20]. If increased cellular cholesterol content exerts its protective activity at the lysosomes, apoptotic signaling proceeding without lysosomal involvement should not be affected. Indeed, U18666A was shown to only protect from cell death induced by agents that signal apoptosis via LMP [21].

In NPC disease, all cells accumulate cholesterol in their lysosomes, but the major clinical symptoms are due to neuronal dysfunction. Therefore, we investigated the effect of U18666A-induced cholesterol accumulation on apoptosis sensitivity in rat cortical neurons. In contrast to a previously published study [29], U18666A did not affect viability of cortical neurons in our experimental settings. Thus, cholesterol accumulation *per se* is not toxic to neurons, and cholesterol accumulation actually protected neurons from apoptosis induced by MSDH and oxidative stress. These results might seem contradictory, as NPC is a chronic neurodegenerative disease (i.e., associated with neuronal death). The reason for the neuronal vulnerability has not been elucidated, but neurons seem to be particularly susceptible to disturbances of lysosomal function [35], [36], and cholesterol storage in lysosomes induces additional changes in the lysosomal system. In the brains of NPC1^−/−^ mice, increased levels of cathepsins have been demonstrated [37]. Furthermore, in NPC-mutant cells, fusion and fission of late endosomes and lysosomes are reduced [38], and vesicle trafficking is impaired [39], [40]. Although cholesterol accumulation confers protection toward acute stress, it remains likely that the associated additional disturbances in lysosomal function may have deleterious effects in the cell in the long run. Noteworthy, disruption of the lysosomal system is implicated in the development of many neurodegenerative disorders that also have a connection to altered cholesterol homeostasis, such as Alzheimer's, Parkinson's and Huntington's diseases [35]. These disorders are characterized by selective vulnerability of specific brain areas to neurodegeneration and oxidative stress [41]. Interestingly, in cells adapted to chronic oxidative stress, resistance was associated with intracellular cholesterol accumulation. Analysis of brain tissue reveals that stress-resistant cells *in vitro* showed similar features to the less vulnerable cerebellum in mice, whereas stress-sensitive cells resembled the highly sensitive hippocampal area [42]. These results highlight the possibility that alterations in membrane cholesterol composition may be at least partly involved in the responses allowing neurons to cope with prolonged stress.

Several factors have been suggested to be involved in the regulation of lysosomal stability, such as the lipid composition of the lysosomal membrane as well as lysosomal membrane proteins. Our results demonstrate that the manipulation of lysosomal cholesterol content can be used to modify apoptosis sensitivity. Our data indicate that short-term lysosomal cholesterol modulation might be used as a therapeutic strategy for conditions associated with accelerated or repressed apoptosis.

## Materials and Methods

### Ethics statement

The animal experiments were approved by the Ethics Committee for Animal Research at Linköping University (permit number 101/08).

### Cells and culture conditions

Wt (GM05659) and NPC1-mutant (GM18436) human fibroblasts (passages 12–24; Coriell Institute, Camden, NJ, USA) were cultured in Eagle's minimum essential medium supplemented with 10% fetal calf serum, 2 mM glutamine, 50 IU/ml penicillin-G and 50 μg/ml streptomycin (all from Gibco, Paisley, UK). Cells were incubated in humidified air with 5% CO_2_ at 37°C and were subcultured once a week. Primary cultures of newborn rat neurons were obtained essentially as described elsewhere [43]. In short, newborn Sprauge-Dawley rats (Taconic Europe, Lilla Skensved, Denmark) were decapitated and the cortex dissected. The cortex tissue was sieved through a nylon mesh (80 μm) into Neurobasal A medium, 50 IU/ml penicillin, 50 μg/ml streptomycin, 0.5 mM glutamine and 2% B27 supplement, with addition of 5 ng/ml β-fibroblast growth factor (β-FGF; β-fibroblast growth factor Life Technologies, Darmstadt, Germany). Cells were plated on poly-L-lysine coated surface at a density of 100000 cells/cm^2^. After 24 h, half of the media was changed to receive a final concentration of 10 ng/ml β-FGF. Every third day, half of the medium was replaced with fresh media (10 ng/ml β-FGF). Cells were used for experiments at day 12–18. LAMP-1^−/−^, LAMP-2^−/−^, LAMP^null^ and wt mouse embryonic fibroblasts (MEFs) were generated as previously described [30]. The cells were grown in Dulbecco's minimum essential medium containing 50 IU/ml penicillin-G, 50 μg/ml streptomycin and 10% fetal calf serum (all from Gibco). Cells were incubated in humidified air with 5% CO_2_ at 37°C and were subcultured twice a week. Prior to experiments, fibroblasts were trypsinized and seeded at a density that allowed them to reach 80% confluence at the time of apoptosis induction. Cells were pre-treated with U18666A (0.25-3 μg/ml), quinacrine (2 μM) and 25-HC (1 μg/ml; all from Sigma-Aldrich, St. Louis, MO, USA) for 48 h. MβCD (400–500 μM; Sigma-Aldrich) was added to cells for 1 h to allow endocytosis and then removed and cells were chased for 24 h. This approach was shown to deplete cholesterol from the lysosomal membrane rather than the plasma membrane [44]. Cells were treated with myriocin (10 μM; Sigma-Aldrich) or vehicle (dimethyl sulfoxide; DMSO) for 48 h before analysis or apoptosis induction.

### Apoptosis induction

Apoptosis was induced by exposing cells to the lysosomotropic detergent MSDH (10–15 μM; kindly provided by Gene M. Dubowchik, Bristol-Myers Squibb, Wallingford, CT, USA), glucose oxidase (GO; 1.6 μg/ml, Sigma-Aldrich) or H_2_O_2_ (570–760 µM; Sigma-Aldrich). The concentrations were optimized to induce apoptosis without necrotic contamination, as judged by morphologic examination of cell cultures. MSDH was added in serum-free medium for 24 h. All drugs (U18666A, quinacrine, 25-HC, MβCD and myriocin) were omitted during the exposure. Cells were exposed to H_2_O_2_ in serum-free medium for 2 h and then incubated for 24 h in complete medium before analysis. GO, an enzyme that catalyzes the oxidation of glucose and generates H_2_O_2_, was freshly prepared prior to each experiment (1 mg/ml in 50 mM sodium acetate, pH 5.1). Neurons were exposed to GO in complete medium for 1 h, and then the medium was exchanged to serum free medium for 24 h.

### Viability analysis

After treatment, cell cultures were morphologically examined in a phase contrast microscope and viability was measured using the 3-(4,5-dimethylthiazol-2-yl)-2,5-diphenyltetrazolium bromide (MTT; Calbiochem, San Diego, CA, USA) reduction assay. Cells were incubated with 0.25 mg/ml MTT for 2h at 37°C. The MTT solution was then removed and the formazan product dissolved in DMSO. The absorbance was measured at 550 nm. In addition, the amount of surviving and thus attached cells was determined using crystal violet staining. Cells were fixed in 4% paraformaldehyde for 20 min, followed by 0.04% crystal violet staining for 20 min at room temperature. The plates were washed thoroughly by dipping in H_2_O and subsequently air-dried. Samples were then solubilized in 1% Sodium dodecyl sulfate (SDS) before absorbance was measured at 550 nm. Caspase-3-like activity was analyzed using the substrate Ac-DEVD-AMC (Becton, Dickinson and Company, Franklin Lakes, NJ) according to the manufacturer's instructions. Fluorescence was correlated to protein content.

### Lipid measurements

Unesterified cholesterol content was measured in cell lysates using the Amplex Red Cholesterol Assay Kit (Invitrogen, Paisley, UK), as described by the manufacturer. Cholesterol amount was correlated to protein content. Sphingomyelin content was analyzed according to a previously described method [28].

### Immunocytochemistry

Cells were prepared for immuno-cytochemistry as described elsewhere [20]. Antibodies against LAMP-2 (Southern Biotech, Birmingham, AL, USA), followed by antibodies conjugated to Alexa Fluor (Molecular Probes), were used. To visualize unesterified cholesterol, cells were stained with filipin (125 μg/ml; Sigma-Aldrich) for 1 h at room temperature. Cover slips were washed and mounted using Prolong gold (Invitrogen). Cells were examined using a Nikon Eclipse E600 laser scanning confocal microscope (Nikon, Tokyo, Japan) together with the EZC1 3.7 software (Nikon Instruments, Melville, NY, USA) or a Nikon Eclipse TE2000U microscope (Nikon) with a Bio-Rad Radiance 2100 MP confocal system (Carl Zeiss, Jena, Germany).

### Flow cytometric determination of Lysotracker fluorescence

Cells were stained with 50 nM Lysotracker green-26 (Invitrogen) for 5 min at 37°C and detached by trypsinization. Lysotracker fluorescence was analyzed in a LSR flow cytometer (Becton Dickinson Biosciences, Franklin Lakes, NJ, USA) using a 488 nm argon laser and the resulting fluorescence was detected in the FL1 channel using a 530±28 nm filter. Data from 10000 cells were collected and was analyzed using CellQuest software (Becton Dickinson Biosciences).

### Western blot analysis

Protein separation was performed as described previously [20]. Proteins were blotted onto a nitrocellulose membrane using an iBlot Dry Blotting System (Invitrogen). The following primary antibodies were used: mouse anti-LAMP-2 (1∶1000; Southern Biotech, Birmingham, AL, USA) and mouse anti-glyceraldehyde-3-phosphate dehydro-genase (GAPDH; 1∶5000; Novus Biologicals, Littleton, Co, USA).

### Determination of lysosomal membrane stability

To analyze the integrity of lysosomes, photo-oxidation of AO (Gurr, Poole, UK) was employed as described earlier [23]. AO is a metachromatic dye that, when excited by blue light, emits red fluorescence when highly concentrated inside lysosomes and green fluorescence when diluted in the cytosol [26]. Cells seeded on coverslips were incubated with AO (2 µg/ml) for 15 min at 37°C, washed with phosphate buffered saline (PBS), and placed on the stand of a Nikon Eclipse E600 laser scanning confocal microscope. AO was excited using a 488 nm light from a 100-mW diode laser, and loss of lysosomal proton gradient was followed by capturing laser scanning micrographs every 330 ms in a channel defined by bandpass filters for 495–555 nm. Green fluorescence intensity in pre-defined areas was subsequently analyzed using Volocity (PerkinElmer, Waltham, MA, USA) and plotted. The loss of lysosomal integrity was determined as the lag time from the start of blue laser irradiation until the rupture of lysosomes induced an increase of green fluorescence in the cytosol (Figure 3E).

### Statistical analysis

All experiments were repeated at least three times and the results are presented as the means and standard deviations of independent samples. Data were statistically evaluated using a nonparametric Kruskal-Wallis test, followed by Mann-Whitney U test for comparison of two groups. P values ≤0.05 were considered to be significant and marked with an asterisk in figures.

## Supporting Information

Figure S1 Viability of human fibroblasts after MSDH exposure as assessed by crystal violet staining. Human wt fibroblasts were treated with U18666A or quinacrine to induce cholesterol accumulation, and NPC1-mutant fibroblasts were treated with methyl-β-cyclodextrin (MβCD) or 25-hydroxy cholesterol (25-HC) to revert cholesterol storage. Viability of cultures assessed by crystal violet staining (n = 4). Viability is expressed as percentage of untreated cultures. Data are presented as the mean ± SD, * p≤0.05.
(TIF)

Figure S2 Measurement of cholesterol content in primary neuronal cultures. Cultures of rat neurons were treated with U18666A (0.5–3 μg/ml, 48 h) and the unesterified cholesterol content was measured (n = 3). Data are presented as the mean ± SD, ns; non-significant.
(TIF)
