# Supplementary figures and images for: Sensitivity to Lysosome-Dependent Cell Death Is Directly Regulated by Lysosomal Cholesterol Content
Source: PLoS One. 2012 Nov 16;7(11):e50262. doi: 10.1371/journal.pone.0050262 (PMC3500374; doi:10.1371/journal.pone.0050262)

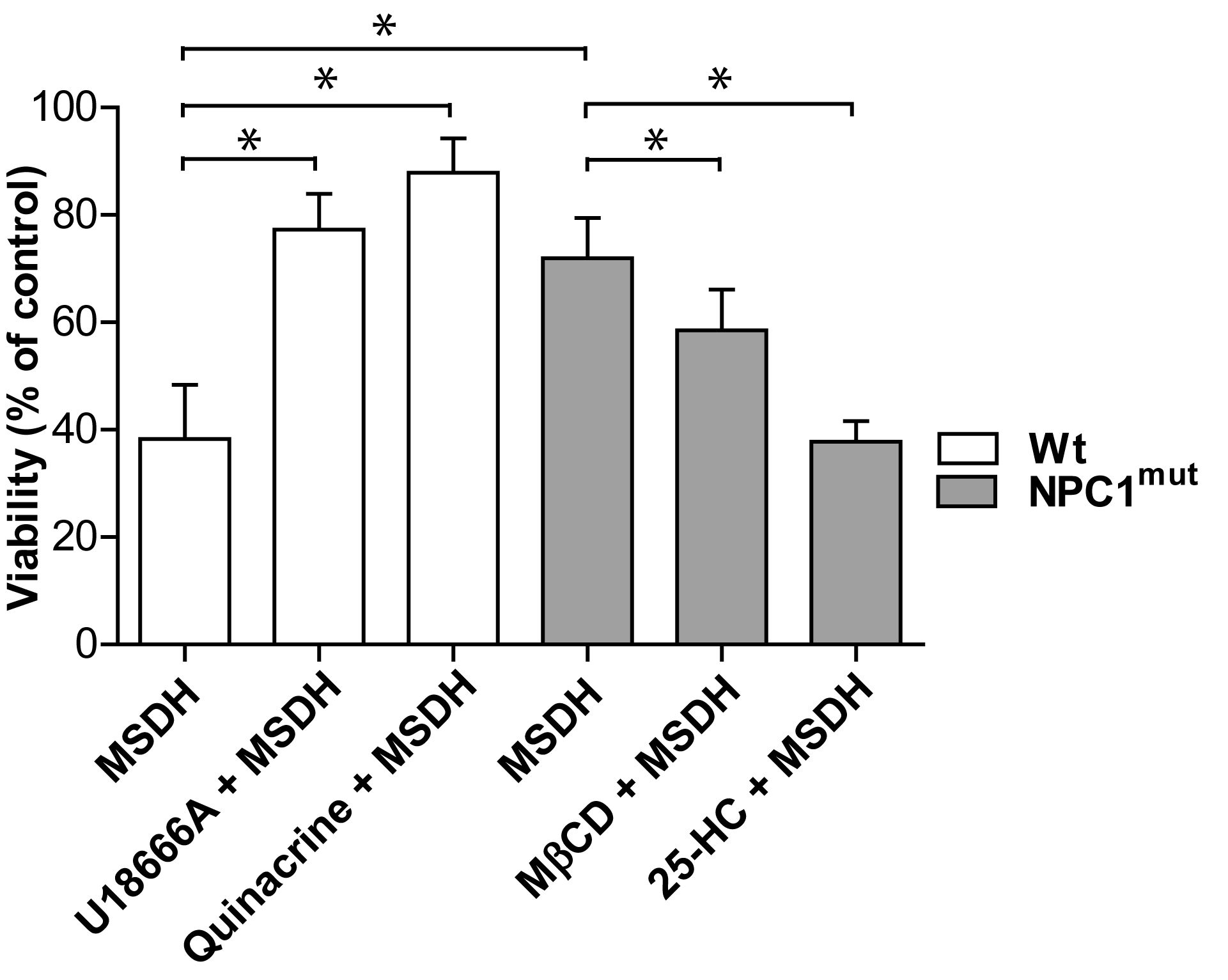

Supplement: Figure S1 — Viability of human fibroblasts after MSDH exposure as assessed by crystal violet staining. Human wt fibroblasts were treated with U18666A or quinacrine to induce cholesterol accumulation, and NPC1-mutant fibroblasts were treated with methyl-β-cyclodextrin (MβCD) or 25-hydroxy cholesterol (25-HC) to revert cholesterol storage. Viability of cultures assessed by crystal violet staining (n = 4). Viability is expressed as percentage of untreated cultures. Data are presented as the mean ± SD, * p≤0.05. (TIF) [file pone.0050262.s001.tif]

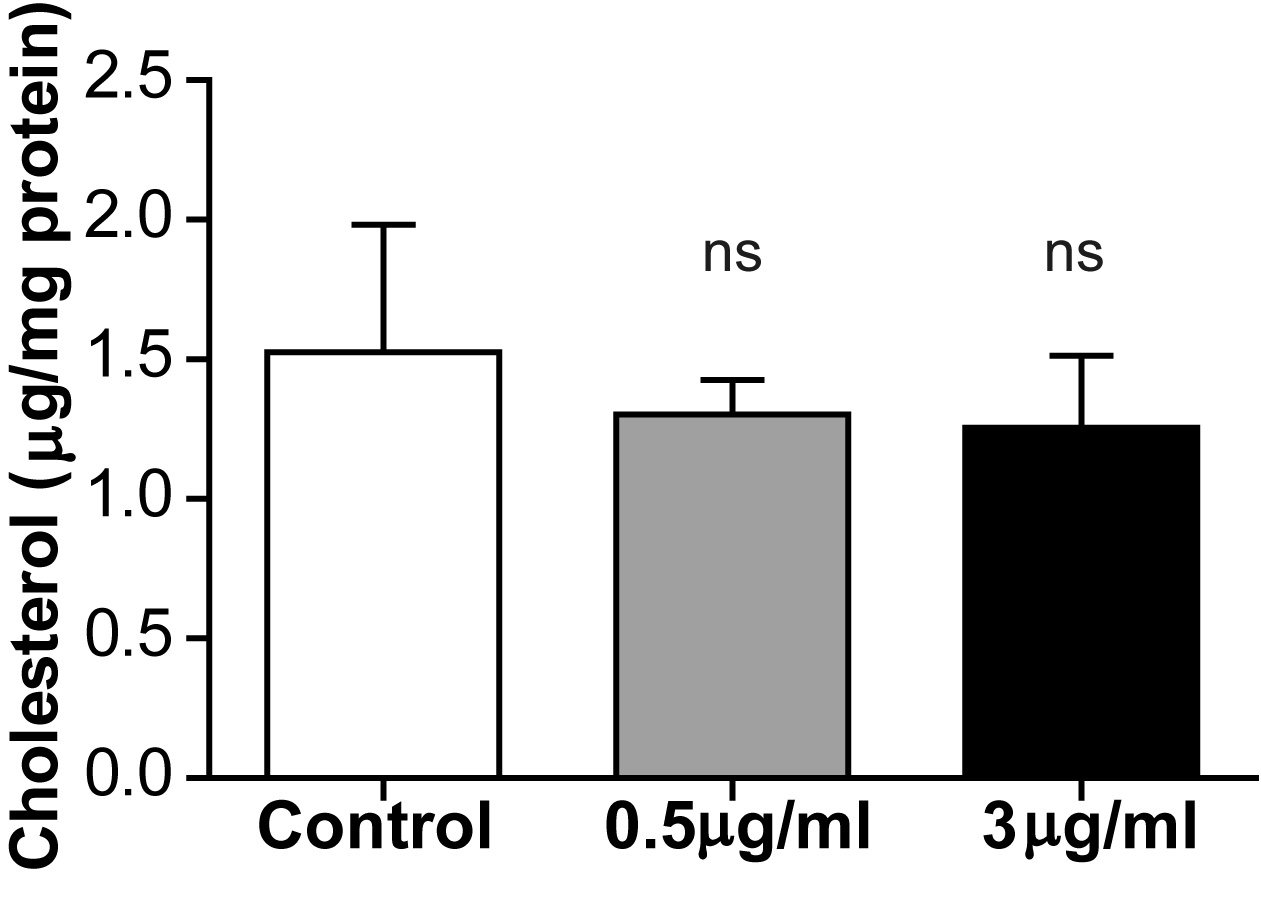

Supplement: Figure S2 — Measurement of cholesterol content in primary neuronal cultures. Cultures of rat neurons were treated with U18666A (0.5–3 μg/ml, 48 h) and the unesterified cholesterol content was measured (n = 3). Data are presented as the mean ± SD, ns; non-significant. (TIF) [file pone.0050262.s002.tif]
